# Intensive Care Medicine in Portugal

**DOI:** 10.62675/2965-2774.20250344

**Published:** 2025-02-10

**Authors:** Rui Moreno, Andrew Rhodes, Ederlon Rezende

**Affiliations:** 1 Hospital de São José Unidade Local de Saúde São José Lisboa Portugal Hospital de São José, Unidade Local de Saúde São José - Lisboa, Portugal.; 2 Faculdade de Ciências Medicas de Lisboa Nova Medical School Lisboa Portugal Faculdade de Ciências Medicas de Lisboa, Nova Medical School - Lisboa, Portugal.; 3 Faculdade de Ciências da Saúde Universidade da Beira Interior Covilhã Portugal Faculdade de Ciências da Saúde, Universidade da Beira Interior - Covilhã, Portugal.; 4 St. George’s University Hospitals NHS Foundation Trust St. George’s University of London London United Kingdom Adult Critical Care, St. George’s University Hospitals NHS Foundation Trust, St. George’s University of London - London, United Kingdom.; 5 Department of Intensive Care Hospital do Servidor Público Estadual ¨Francisco Morato de Oliveira Instituto de Assistência Médica ao Servidor Público Estadual São Paulo SP Brasil Department of Intensive Care, Hospital do Servidor Público Estadual ¨Francisco Morato de Oliveira, Instituto de Assistência Médica ao Servidor Público Estadual - São Paulo (SP), Brasil.

Healthcare expenditures are rising rapidly in many developed countries, and debates are being focused on coping with the rising demand. The ability to address the increasing number of older individuals who often present with multiple chronic conditions differs significantly from what most healthcare systems were designed to support. There is a growing need to prevent illnesses rather than treat them as well as to enable systems to better manage complex, frail patients. There is also increasing recognition in many settings that the traditional model of stand-alone hospitals may not be optimal for future healthcare needs, and groups (or systems) of healthcare providers are coming together in networks, referential axes or accountable organizations to share resources and better deploy assets for the populations they serve. Like many other countries, hospitals and primary healthcare budgets and oversight are being consolidated in Portugal. This has implications for Intensive Care Medicine (ICM), which must rethink and review what it means for this still-young specialty.

Intensive care units (ICUs) have been part of the healthcare system in Portugal since 1959, although the ICM specialty was only described in 1989.^(1)^ The recognition of ICM as a formal multidisciplinary supraspecialty requiring specific training soon followed in the early 2000s. At that time, Portugal had the lowest number of ICU beds in Europe (4.2/100,000 capita).^(2)^In recent years (2015), adult ICM has been recognized as a primary supraspecialty,^(3)^ followed by official regulation of the training program in 2016,^(4)^ with the first residents starting their 5 years of training shortly after. Since then, more intensivists have come from the primary training model, with the multiprofessional training model (now closed) disappearing with time and with the retirement of professionals.

The COVID-19 pandemic forced many changes in specialty and training programs. Even before the pandemic, in 2016, it was realized that better service delivery could be achieved by networking ICUs nationwide to provide operational support and learn and improve from each other.^(5)^ This was accelerated in 2020, during the first wave of COVID-19,^(6)^ when the necessities of the pandemic surge response required these networks to function adequately. Moreover, owing to the requirement to increase capacity, cope with the pandemic, and network across the country, the first tranche of ICM-trained specialists started to expand the workforce, and they have been crucial in this process.

Rapid development in networks has been required because not all ICUs are equivalent. It is not possible to maintain all specialist skills in every hospital, either because of the requirements for sufficient volumes of activity to maintain skills or because of the constraints imposed by a still small workforce.^(7)^ As the networks and axes of reference have grown, however, the need to move patients safely between the constituent parts of each system has become a critical issue. The current system is amateur and inadequate, resulting in the need to create a professional interhospital transfer system for critically ill patients who can cope with the challenges of secondary and tertiary patient transfer.

The new ICM training program is ideally positioned to support these developments. Primary ICU training programs now provide increased numbers of skilled and professional staff, ideally placed to help support the needs of hospitals and pre- and posthospital care. This effort should be supported quickly, as noted earlier this month in the Urgency and Emergency Medicine as a Primary Specialty (*Ordem dos Médicos, Regulamento* n° 1223/2024 from October 24 published in the *Diário da República, 2ª série* n° 207 from 10-24-2024). On the mainland, challenges persist, with only five hospitals with certified helipads and a disjointed transport system. This workforce has all the skills needed to provide the interfacility transfer support that is required to make the networks function well, even in the geographically isolated parts of our country, such as the archipelagos of Madeira and Açores, where owing to the help of the Air Force, the situation is better organized.

These changes have significantly improved the specialty situation. In 2020, before the COVID-19 pandemic, Martins et al.^(8)^published a summary of ICU resources in Portugal. They reported that the number of ICU beds increased from 4.2 to 6.5/100,000 inhabitants. However, high occupancy rates of over 90% have been reported, whereas the optimal rates are between 70% and 75%,^(9)^ depending on the case mix and workload.^(10,11)^

At the end of the pandemic, and according to the authors of the manuscript published in this issue of Critical Care Science,^(12)^ Portugal had 7.5 intensivists per 100,000 inhabitants). The number of ICU beds peaked in 2022 at 8.8/100,000 and then stabilized at 8.0. This post-COVID-19 reduction followed a similar pattern as that witnessed in many other countries. This placed Portugal again below the Organisation for Economic Co-operation and Development (OECD) median (12/100,000 inhabitants) and created a gap between demand and capacity, with approximately half of ICU department directors acknowledging that they needed more staffed ICU beds.^(12)^ The increase in the number of intensivists resulting from the creation of the primary specialty allowed the field of ICM to expand its portfolio with its new skilled workforce supporting the care of critically ill patients in other areas outside of the ICU, including the prehospital emergency system, the emergency department, intrahospital emergency teams, and follow-up clinics. It is becoming, as desired by its founders, the crucial force around which all the needs of critically ill patients will turn around, coordinating the overall delivery of specialized care in collaboration with other specialties. This may allow for the reactivation and staffing of closed ICU beds, which is critical to balancing supply and demand, reducing care gaps, and preventing staff burnout.^(13)^

There is now a need to reopen and restaff these closed beds, bringing some of the staff with specialty training back into the ICM environment and supporting the current workforce in developing and maintaining a work‒life balance conducive to a long-term career. Creating this additional capacity, over and above what is currently available, is critical to avoid relearning the lessons from the recent pandemic and to provide sufficient breathing space to cope with future problems.

Additionally, low salaries and conditions push many doctors (40 - 50%) and nurses^(14,15)^ to emigrate or move to private practice.^(15-18)^ With one of the highest ratios of doctors per 1,000 inhabitants in the OECD (5.6 per 1,000),^(19)^ Portugal struggles, as only 30% of its 70,481 MDs work locally. Many doctors have emigrated or retired, and while foreign MDs (4,770 in 2024) help, they lack recognized specialty training. The departure of new graduates (30 - 40%) further exacerbates this challenge.^(18)^ Addressing these issues is crucial to ensure timely and adequate critical care. When the executive director of the National Healthcare System appointed a new commission to propose a revision of the network and referral axis,^(20)^ the authors of this manuscript^(12)^ provided precise data for informed decision-making. Accurate estimates are needed to improve the system and prevent care disparities, ensuring access to appropriate care for all critically ill patients. Now is the time for politics to decide, considering the population’s needs more than the existing lobbies do.
